# Real-World Data in Children with Spinal Muscular Atrophy Type 1 on Long-Term Ventilation Receiving Gene Therapy: A Prospective Cohort Study

**DOI:** 10.3390/arm92050032

**Published:** 2024-08-28

**Authors:** Mohammad Ala’ Alajjuri, Rania Abusamra, Vivek Mundada, Omendra Narayan

**Affiliations:** 1 College of Medicine, University of Sharjah, Sharjah, United Arab Emirates; onarayan@ahdubai.com; 2 Dubai Health, Dubai, United Arab Emirates; 3 Department of Pediatric Pulmonology, Mediclinic City Hospital, Dubai, United Arab Emirates; raniaabusamra@gmail.com; 4 Department of Pediatric Neuroscience, Aster DM Healthcare, Medcare Women and Children Hospital, Dubai, United Arab Emirates; vivek.mundada@medcarehospital.com; 5 Department of Pediatric Pulmonology, American Hospital, Dubai, United Arab Emirates

**Keywords:** spinal muscular atrophy, SMA, onasemnogene abeparvovec, Zolgensma, tracheostomy, invasive ventilation, long-term ventilation

## Abstract

**Highlights:**

**What are the main findings?**

- Gene therapy at a late age was safe and effective in resolving paradoxical breathing, improving cough ability, reducing airway secretions, and enhancing CHOP-INTEND scores over a short-term period in patients with SMA-1 ventilated via tracheostomy.
- The clinical assessment and management implemented pre-gene therapy were effective in safely weaning patients for at least 8 h off ventilator per day.

**What is the implication of the main finding?**

- Older patients with SMA-1 still have a chance for improvement if presented late for gene therapy.
- In conjunction with gene therapy, high-quality clinical care is beneficial and should be paired with gene therapy management.

**Abstract:**

Patients with spinal muscular atrophy type 1 (SMA-1) requiring invasive ventilation can be eligible for gene therapy if they tolerate at least 8 h off ventilation per day. We aimed to assess the short-term safety and efficacy of gene therapy (onasemnogene abeparvovec; Zolgensma) on respiratory function in SMA-1 patients ventilated via tracheostomy pre-gene therapy. A prospective cohort study included 22 patients. Patients were weaned off ventilation for at least 8 h daily by optimizing ventilator settings and duration, using cough augmentation, managing excessive airway secretions, enhancing nutrition, screening for respiratory bacterial colonization, and treating infections. Gene therapy was administered at a median age of 26 (Q1: 18, Q3: 43) months with a mean follow-up period of 7.64 (SD: 6.50) months. Gene therapy was safe and effective in resolving paradoxical breathing, improving cough ability, reducing airway secretions, and enhancing CHOP-INTEND scores. The clinical assessment and management implemented pre-gene therapy were effective in safely weaning patients for at least 8 h off ventilation daily. Gene therapy at a late age was safe and effective over the short-term period; however, long-term follow-up is recommended. In conjunction with gene therapy, high-quality clinical care is beneficial and should be paired with gene therapy.

## 1. Introduction

### 1.1. Definition

Spinal muscular atrophy (SMA) is a rare congenital autosomal recessive genetic disorder characterized by progressive symmetric degeneration of alpha motor neurons in the anterior horn cells in the spinal cord and brainstem, resulting in muscle weakness and atrophy [1,2,3]. Disease incidence ranges from 1 per 6000 to 11,000 live births, accounting for the second most common cause of genetic death [4]. Incidence is 40 times higher in the Middle East than in the Western Pacific region, attributed to higher consanguinity rates [5]. Nearly 96% of SMA cases arise from deletions or mutations in the survival motor neuron 1, telomeric (*SMN1*) gene on chromosome 5q13.2, resulting in reduced production of survival motor neuron protein [6]. Disease severity ranges from mild to severe, depending on the allelic form mutated in the *SMN1* gene and the number of *SMN2* gene copies that are responsible for producing 10% to 15% of SMN protein [7]. *SMN2* gene copies range from 0 to 8, reflecting the amount of survival motor neuron protein produced [8,9].

### 1.2. Clinical Features

The respiratory and neurological systems are the most affected body systems by the disease; patients present with drooling, hypotonia, dysphagia, gastroesophageal reflux, and malnutrition [1,2,3,10]. Therefore, a multidisciplinary team is needed to assess and manage the neuromuscular, musculoskeletal, swallowing, and nutritional aspects [11]. Cough augmentation helps in preventing hospitalizations and decreases hospital stays from respiratory infections. Sleep studies are required to assess patients’ oxygenation status, ventilation, and respiratory support requirements to modify ventilation or wean patients off ventilatory support [12]. The thoracic-to-head circumference ratio is a new measure used to evaluate SMA-1 severity and prognosis, in which patients with a ratio of less than 0.85 die within 3 months [13]. Respiratory failure is the leading cause of death; thus, patients require long-term respiratory support [14]. Patients usually become dependent on ventilation before the age of 1 year [15]. However, continuous reduction in respiratory function is still ongoing with supportive care [2]. Long-term ventilation is defined as either ventilation requirement for 16 h or more per day for at least 14 successive days unrelated to acute reversible disease or perioperative use, or tracheostomy placement [16]. Without supportive care, the death rate is 68% within 2 years and 82% within 4 years of age. However, with respiratory and nutritional support, patients achieve higher survival rates, leading to a nearly 30% reduction in mortality at 2 years of age [17]. Home-invasive and non-invasive ventilation reduces hospitalization rates and hypoxic events, leading to a prolonged lifespan and improved quality of life [18].

### 1.3. Gene Therapy

Gene therapy (onasemnogene abeparvovec, Zolgensma) was approved by the United States Food and Drug Administration in May 2019 [15]. It consists of an adeno-associated viral vector that delivers the functional *SMN1* gene to motor neurons and peripheral tissues through a single intravenous infusion, resulting in increased protein formation and prolonged survival [7,12]. Clinical trials have shown that gene therapy infusion at an earlier age has better outcomes [19,20]. The expected survival after gene therapy is 37.2 years of life [21].

### 1.4. Aims

This study was conducted to assess, in a single-center, real-world data about the short-term safety and efficacy of gene therapy on respiratory function in patients with SMA-1 who are on long-term ventilation via tracheostomy before gene therapy infusion.

## 2. Materials and Methods

### 2.1. Design and Setting

A prospective cohort study was conducted to assess the short-term safety and efficacy of gene therapy (onasemnogene abeparvovec; Zolgensma) on respiratory function in SMA-1 patients who are on long-term ventilation via tracheostomy. Patients were referred to the long-term ventilation (LTV) clinic as part of the multidisciplinary team approach for pre-gene therapy assessment. Patients had been followed elsewhere until the initial presentation at the LTV clinic. Patients were referred from May 2021 until October 2022.

### 2.2. Study Size

A total of 22 patients were included in the study. The study size depended on the number of patients referred to our clinic and met the inclusion criteria. Inclusion criteria included patients with genetically confirmed SMA-1, ventilated via tracheostomy, and eligible to receive gene therapy. Patients diagnosed with other subtypes of SMA, not ventilated or ventilated via modalities other than tracheostomy, or ineligible to receive gene therapy were excluded from the study. Patients must tolerate at least 8 h off ventilation per day without compromising gas exchange and work of breathing to be eligible to receive gene therapy, according to the Global Managed Access Program criteria [22]. The European Medicines Agencies approved gene therapy for patients weighing up to 21 kg without age limits [23].

### 2.3. Assessment and Management

All patients were evaluated by the multidisciplinary team, which involved a pediatric pulmonologist, pediatric neurologist, pediatric orthopedics, spine surgeon, pediatric gastroenterologist, physiotherapist, rehabilitation clinician, occupational, speech, and feeding therapists. Patients were assessed subjectively and objectively by polysomnography and barium swallow studies to gradually and safely wean patients off ventilation for a minimum of 8 h per day. Only two pediatric pulmonologists assessed patients’ respiratory function, and the same pediatric pulmonologist evaluated the same patient before and after gene therapy infusion. Objective lung function assessment like a spirometry test could not be performed, as all our patients were ventilated via tracheostomy. Safe and effective gas exchange was denoted by oxygen saturation (SpO2) > 94% without respiratory distress. Our patients were assessed for (1) nutritional status; (2) thoracic-to-head circumference ratio; (3) spine deformity using spine X-ray; (4) work of breathing while off ventilation, including paradoxical breathing, chest expansion, and bell-shaped chest; (5) optimizing ventilator settings and duration—patients were ventilated using spontaneous trigger mode with high sensitivity trigger and safety tidal volume with back-up respiratory rate to ensure adequate volume without increasing the risk of barotrauma; (6) apnea–hypopnea index, SpO_2_ using pulse oximeter, total rapid eye movement (REM) stage percentage of total sleep time, arousal index, and end-tidal carbon dioxide (EtCO_2_) by polysomnography; (7) cough ability and cough augmentation requirement using sodium chloride nebulization and passive expiratory vibrations using mechanical insufflation–exsufflation (MI-E) devices; (8) presence of airway secretions; (9) screening for respiratory bacterial colonization and treating infections; (10) swallowing ability by barium swallow study; and (11) Children’s Hospital of Philadelphia Infant Test of Neuromuscular Disorders (CHOP-INTEND) score. Variables were assessed and compared before and after gene therapy to assess its efficacy.

Patients who had unsatisfactory gas exchange or depended on the ventilator for more than 16 h per day before gene therapy were assessed for modifiable factors, including optimizing ventilator settings and duration, using cough augmentation for airway clearance and enhancing lung volume for better cough ability, suctioning and introducing hyoscine hydrobromide patches for managing excessive airway secretions, enhancing nutrition, and administering antibiotics based on sensitivity for positive airway bacterial colonization or infections. Patients with Pseudomonas-positive tracheal aspirate cultures were treated with oral ciprofloxacin for 2 weeks and nebulized tobramycin or colistin for 4 weeks before receiving prednisolone with gene therapy. Sick patients were admitted to the pediatric intensive care unit for intravenous antibiotics.

### 2.4. Gene Therapy Infusion

The duration from post-referral to the clinic until gene therapy infusion depended on patients’ ability to safely tolerate at least 8 h off ventilation per day, along with treating respiratory infections or malnutrition. A 1-month washout period was given between the last dose of nusinersen and gene therapy. Liver function tests, platelet count, coagulation profile, and troponin I were assessed before and after gene therapy to assess its safety.

### 2.5. Data Collection and Analysis

Data were collected from hospitals’ electronic medical records and teleconsultations from July 2022 until June 2023. Data were coded and analyzed using the Statistics Package for the Social Sciences software version 25. Descriptive statistics were conducted, including frequency, percentage, mean, standard deviation (SD), median, first quartile (Q1), and third quartile (Q3). The data were assessed for skewness; the mean and SD were used for normally distributed data, and the median with Q1 and Q3 were used for skewed data. McNemar and Wilcoxon tests were conducted on categorical and continuous variables, respectively, to assess the relationship between variables. A *p*-value ≤ 0.05 was considered statistically significant.

### 2.6. Follow-Up Period

Patients were monitored weekly over a mean period of 7.64 (SD: 6.50) months after gene therapy. Patients had clinical assessments and blood tests during their visits, which were conducted in person and through teleconsultations through online video meetings. No patients were withdrawn or excluded during the study.

### 2.7. Ethical Approval

Written informed consent was obtained from parents to participate in the study, and ethical approval was obtained from the Ethics & Research Committee, approval reference number: MWCH/ETHICS/003.

## 3. Results

### 3.1. Demographics and Characteristics

Gender was equally distributed, and 11 patients (50.0%) were males. Most of our subjects were Turkish (17 patients, 77.3%), and the remaining 5 patients were Russian, Filipino, Nepalese, and Indian. All patients had *SMN1* gene mutations on chromosome 5q13.2 with two copies of the *SMN2* gene. Twenty patients (90.9%) received nusinersen before gene therapy at a median age of 8.5 (Q1: 7, Q3: 9) months at the first dose. On average, each patient received 6.59 (SD: 3.29) nusinersen injections.

Patients were diagnosed at a mean age of 4.39 (SD: 1.89) months. Patients had tracheostomy placement at an average age of 11.5 (SD: 5.75) months. The mean age at presentation to our clinic was 29.2 (SD: 17.1) months. Patients received gene therapy at a median age of 26 (Q1: 18, Q3: 43) months. The median period from the first presentation to the LTV clinic until gene therapy infusion was 1 (Q1: 1, Q3: 2) month.

Regarding nutritional status, the mean weight at gene therapy infusion was 10.3 (SD: 2.32) kg. Thirteen patients (59.1%) were fed via a percutaneous endoscopic gastrostomy (PEG) tube before presentation to our clinic. Nine patients (40.9%) were fed via a nasogastric tube, of whom three patients required PEG tube placement after presentation due to micro-aspiration and failure to thrive. The median age at PEG tube placement was 15 (Q1: 10, Q3: 19) months. As for spine deformities, scoliosis was present in 11 patients (50.0%), of whom two patients (9.09%) had concomitant pectus excavatum. The thoracic-to-head circumference ratio was assessed in 12 patients; the mean ratio was 1.07 (SD: 0.12).

### 3.2. Gene Therapy Safety

According to recommendations, all patients were started on oral prednisolone 1 mg/kg 1 day before gene therapy infusion while monitoring liver functions and platelet count [24]. Gene therapy was well tolerated clinically in our patients. Liver function tests, platelet count, coagulation profile, and troponin I levels were normal post-gene therapy.

### 3.3. Work of Breathing

Paradoxical breathing was assessed in 18 patients; 13 patients (72.2%) had paradoxical breathing pre-gene therapy compared with 4 patients (22.2%) post-gene therapy (*p* = 0.004). Chest expansion was assessed in 17 patients; 9 patients (52.9%) had reduced chest expansion pre-gene therapy compared with 2 patients (11.8%) post-gene therapy (*p* = 0.109). Bell-shaped chest was assessed in 15 patients; 7 patients (46.7%) had bell-shaped chest pre-gene therapy compared with 3 patients (20.0%) post-gene therapy (*p* = 0.125).

### 3.4. Time off Ventilator

Time off the ventilator per day was assessed for all patients at presentation, before, and after gene therapy.

*A. At presentation*

Five patients (22.7%) were fully dependent on ventilation around the clock, eleven patients (50.0%) could not tolerate a minimum of 8 h off ventilation per day, and six patients (27.3%) were tolerating a minimum of 8 h off ventilation per day. The median hours off ventilation per day was 3.5 (Q1: 1.5, Q3: 10) hours.

*B. Before gene therapy*

All patients at the time of gene therapy infusion managed to keep off ventilation for at least 8 h per day. The median hours off ventilation per day was 8 (Q1: 8, Q3: 10) hours.

*C. After gene therapy*

Eleven patients (50%) had reduced hours on ventilation; five patients (22.7%) had no changes, and six patients (27.3%) had increased hours on ventilation. One patient (4.55%) was completely weaned off the ventilator 8 months after gene therapy. The median hours off ventilation per day was 10.5 (Q1: 8, Q3: 13) hours compared with 8 (Q1: 8, Q3: 10) hours before gene therapy (*p* = 0.06).

**Figure 1:**
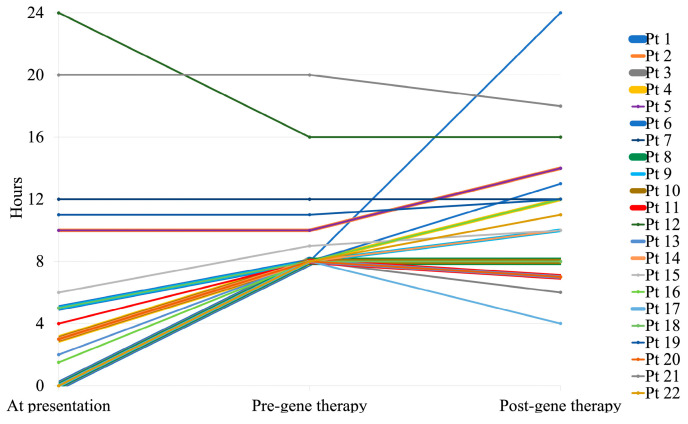
Hours off ventilation per day at presentation, before, and after gene therapy. Pt: Patient.

**Figure 2:**
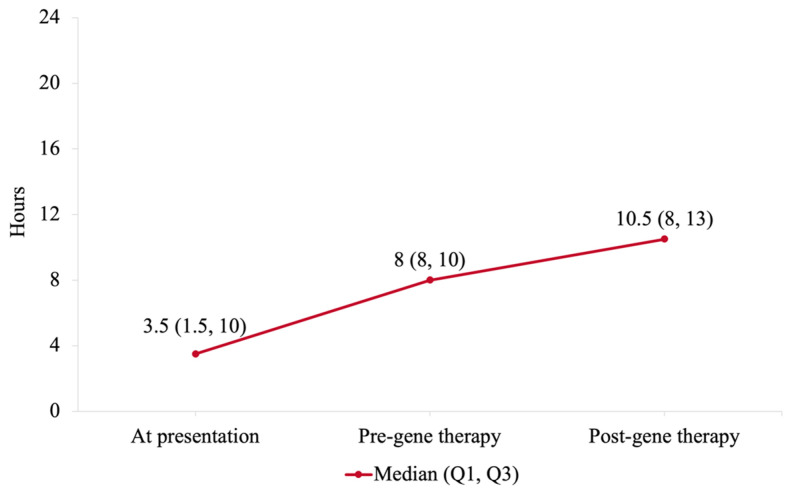
The median (Q1, Q3) hours off ventilation per day at presentation, before, and after gene therapy.

Hours off ventilation per day for the 22 patients at presentation, before, and after gene therapy are delineated in Figure 1. The median (Q1, Q3) hours off ventilation per day at presentation, before, and after gene therapy are shown in Figure 2.

### 3.5. Ventilator Settings

Pressure support and respiratory rate were assessed in 19 patients; the mean pressure support was 10.7 (SD: 3.45) cm H_2_O pre-gene therapy compared with 10.1 (SD: 4.03) cm H_2_O post-gene therapy (*p* = 0.195). The mean respiratory rate was 20.1 (SD: 2.45) breaths per minute pre-gene therapy compared with 19.2 (SD: 2.70) breaths per minute post-gene therapy (*p =* 0.151).

### 3.6. Polysomnography Assessment

Polysomnography was conducted on 12 patients (54.5%) before gene therapy while on ventilatory support, except for one patient who was completely weaned off the ventilator several months before gene therapy. Ten patients (45.5%) could not undergo the test due to financial burden. Patients had a mean apnea–hypopnea index of 3.21 (SD: 2.84) events per hour, a mean SpO2 of 96.6% (SD: 1.08), and a mean lowest SpO2 of 91.8% (SD: 3.01). The total REM stage percentage of total sleep time was 20.0% (SD: 7.28), and the median arousal index was 9.85 (Q1: 8.25, Q3: 17.0) events per hour. EtCO2 was assessed in 10 patients, the baseline EtCO2 mean was 32.7 (SD: 4.90) mmHg, and the mean highest EtCO2 was 41.9 (SD: 3.76) mmHg.

As a result, six patients (50.0%) required ventilator settings adjustment, one patient (8.33%) required less duration on a ventilator, one patient (8.33%) required ventilation initiation due to ineffective gas exchange, and four patients (33.3%) did not require any modifications.

### 3.7. Cough Ability and Augmentation

Cough ability was assessed in 19 patients, 5 patients (26.3%) had preserved cough ability pre-gene therapy compared with 15 patients (78.9%) post-gene therapy (*p* = 0.002). Cough augmentation requirement and frequency per day using MI-E devices were assessed in 17 patients. Fifteen patients (88.2%) required MI-E devices pre-gene therapy compared with sixteen patients (84.2%) post-gene therapy (*p* = 1.00). The mean MI-E usage per day was 2.76 (SD: 1.44) times per day pre-gene therapy compared with a mean of 2.35 (SD: 0.93) times per day post-gene therapy (*p* = 0.191).

### 3.8. Airway Secretions

Tracheal secretions and oropharyngeal drooling were assessed in all patients. All patients had tracheal secretions pre-gene therapy compared with 12 patients (45.5%) post-gene therapy (*p* = 0.002). Twenty-one patients (95.5%) had oropharyngeal drooling pre-gene therapy compared with ten patients (54.5%) post-gene therapy (*p =* 0.001).

Tracheal secretions suctioning requirement per day was assessed in 14 patients; patients had a median of 11 (Q1: 5, Q3: 15) suctions per day pre-gene therapy compared with a mean of 10.1 (SD: 7.73) suctions per day post-gene therapy (*p* = 0.196). Hyoscine hydrobromide patch usage was assessed in 21 patients; 18 patients (85.7%) required patches pre-gene therapy compared with 16 patients (76.2%) post-gene therapy (*p* = 0.625).

### 3.9. Bacterial Colonization and Infections

Several microorganism species were cultured from tracheostomy aspirates from 19 patients; 17 patients (89.5%) had positive results. *Pseudomonas aeruginosa* was positive in all patients, followed by *Staphylococcus aureus*, which was positive in seven patients (41.2%).

Antibiotic requirements for respiratory bacterial colonization or infections and hospital admission rates due to respiratory infections were assessed in 21 patients (95.5%). Sixteen patients (76.2%) required antibiotics pre-gene therapy compared with ten patients (45.5%) post-gene therapy (*p* = 0.07), and four patients (19.0%) were admitted due to respiratory infections pre-gene therapy compared with seven patients (33.3%) post-gene therapy (*p* = 0.508).

### 3.10. Swallowing Ability

Swallowing ability was assessed in all patients; two patients (9.52%) were able to swallow pre-gene therapy compared with six patients (27.3%) post-gene therapy (*p* = 0.063).

### 3.11. Motor Assessment

CHOP-INTEND scores were assessed in 10 patients; patients had a mean score of 32.9 (SD: 9.05) pre-gene therapy compared with a mean of 40.1 (SD: 7.84) post-gene therapy (*p* = 0.005).

## 4. Discussion

### 4.1. Key Findings

Gene therapy showed statistically significant improvements in resolving paradoxical breathing, improving cough ability, reducing airway secretions, and enhancing CHOP-INTEND scores over a mean period of 7.64 (SD: 6.50) months post-gene therapy. Even though patients were not weaned off the ventilator post-gene therapy, except for one patient, gene therapy showed efficacy in improving the strength of respiratory muscles. Although clinical improvements were noted in other variables, statistical analysis was insignificant, attributed to the limited number of patients involved in the study.

Gene therapy was administered at a late age with a median age of 26 (Q1: 18, Q3: 43) months due to the late referral to our center for gene therapy with a mean of 29.2 (SD: 17.1) months. However, gene therapy administration was not delayed, as our patients received it after a median period of 1 (Q1: 1, Q3: 2) month post-referral to the LTV clinic. Gene therapy was effective at a late age of administration, indicating that older patients with SMA-1 still have a chance for improvement if presented late for the therapy. Six patients required more hours on ventilator post-gene therapy due to increased airway secretions. Furthermore, hospital admission rates from respiratory infections were higher post-gene therapy; this was attributed to the short assessment period by a median of 1 (Q1: 1, Q3: 2) month from first presentation to the LTV clinic until gene therapy infusion compared with a longer follow-up period post-gene therapy by a mean period of 7.64 (SD: 6.50) months.

The clinical assessment and management implemented at the LTV clinic were effective in safely weaning our patients for at least 8 h off ventilation per day pre-gene therapy. Thus, in conjunction with gene therapy, rigorous, high-quality clinical care is beneficial and should be paired with gene therapy management.

### 4.2. Results Comparison

Clinical data about the short-term safety and efficacy of gene therapy in patients with SMA-1 ventilated via tracheostomy is limited in the literature [12]; thus, this hinders our ability to compare our findings with similar studies.

It was reported that the ventilatory support requirement for SMA patients would not change after gene therapy [2]. However, 11 patients had reduced hours on ventilation, and 1 patient was completely weaned off the ventilator 8 months after gene therapy.

The STR1VE-EU trial involved 32 SMA-1 patients and a control group of 23 SMA-1 patients to study the effects of gene therapy. Gene therapy was administered to patients under 6 months of age, and patients were followed up until the age of 18 months. The results showed that 31 out of 32 patients (97%) did not require permanent ventilation at the age of 14 months compared with 6 out of 23 patients (26%) in the control group [25]. Similarly, the STR1VE-US trial involved 22 SMA-1 patients and a control group of 23 SMA-1 patients to study the effects of gene therapy. Gene therapy was administered to patients less than 6 months of age, and patients were followed up until the age of 18 months. The results revealed that 20 out of 22 patients (91%) did not require permanent ventilation at the age of 14 months compared with 6 out of 23 patients (26%) in the control group [26].

A study presented a case of a patient with SMA-1 ventilated via tracheostomy treated with gene therapy at the age of 9 months; the patient was weaned off the ventilator from 45 min per day pre-gene therapy to 12 h per day 6 months post-gene therapy [15]. A similar study on a patient with SMA-1 ventilated via tracheostomy did not show improvement in time off ventilation post-gene therapy; this was attributed to irreversible motor neuron damage [12]. The discrepancy in the degree of improvement post-gene therapy is suggested to be due to the earlier age at gene therapy infusion, under 6 months and at 9 months in these studies, compared with the late age at gene therapy administration in our patients, median age of 26 months (Q1: 18, Q3: 43). However, this observation opens the field for more research to be implemented to outline other reasons behind this finding.

### 4.3. Limitations

The absence of a control group and the multidisciplinary assessment and management performed at the presentation to the LTV clinic limit our ability to have a proper conclusion about the efficacy of gene therapy. Additionally, the financial burden prevented some patients from having polysomnography assessment, PEG tube placement, and cough assist devices.

## 5. Conclusions

Gene therapy at a late age was safe and effective in resolving paradoxical breathing, improving cough ability, reducing airway secretions, and enhancing CHOP-INTEND scores over a short-term period in patients with SMA-1 ventilated via tracheostomy. The clinical assessment and management implemented at the LTV clinic were effective in safely weaning our patients for at least 8 h off ventilation per day pre-gene therapy. Thus, in conjunction with gene therapy, rigorous, high-quality clinical care is beneficial and should be paired with gene therapy management. We recommend monitoring patients over a longer period to explore the long-term outcomes.

## Data Availability

The datasets used and analyzed in this study are available from the corresponding author upon reasonable request.
